# A longitudinal study of humoral immune responses induced by a 3-dose inactivated COVID-19 vaccine in an observational, prospective cohort

**DOI:** 10.1186/s12865-022-00532-1

**Published:** 2022-11-16

**Authors:** Yue Tao, Mengyin Ma, Fenghua Hu, Ming Li, Yu Geng, Yawen Wan, Minxin Mao, Lin Chen, Ya Shen, Liguo Zhu, Han Shen, Yuxin Chen

**Affiliations:** 1 Department of Laboratory Medicine, Nanjing Drum Tower Hospital Clinical College of Nanjing Medical University, Nanjing, Jiangsu China; 2 Department of Infectious Diseases, Nanjing Drum Tower Hospital Clinical College of Nanjing University of Chinese Medicine, Nanjing, Jiangsu China; 3 Department of Infectious Diseases, Nanjing Drum Tower Hospital Clinical College of Xuzhou Medical University, Xuzhou, Jiangsu China; 4 Jiangsu Provincial Center for Disease Control and Prevention, Nanjing, Jiangsu China

**Keywords:** COVID-19, SARS-CoV-2, Humoral immune responses, Vaccine, Booster

## Abstract

**Background:**

To determine the dynamic SARS-CoV-2 specific antibody levels induced by 3 doses of an inactivated COVID-19 vaccine, CoronaVac. An observational, prospective cohort study was performed with 93 healthy healthcare workers from a tertiary hospital in Nanjing, China. Serum SARS-CoV-2 specific IgM, IgG, and neutralizing antibodies (NAb) were measured at different time points among participants who received 3 doses of inactivated COVID-19 vaccine.

**Results:**

91.3% (85/93) and 100% (72/72) participants showed positive both for SARS-CoV-2 specific IgG and NAb after 2-dose CoronaVac and after 3-dose CoronaVac, respectively. Anti-SARS-CoV-2 IgG responses reached 91.21 (55.66–152.06) AU/mL, and surrogate NAb was 47.60 (25.96–100.81) IU/mL on day 14 after the second dose. Anti-SARS-CoV-2 IgG responses reached 218.29 (167.53–292.16) AU/mL and surrogate NAb was 445.54 (171.54–810.90) IU/mL on day 14 after the third dose. Additionally, SARS-CoV-2 specific surrogate neutralizing antibody titers were highly correlated with serum neutralization activities against Ancestral, Omicron, and Delta strains. Moreover, significantly higher SARS-CoV-2 IgG responses, but not NAb responses, were found in individuals with breakthrough infection when compared to that of 3-dose CoronaVac recipients.

**Conclusions:**

CoronaVac elicited robust SARS-CoV-2 specific humoral responses. Surrogate NAb assay might substitute for pseudovirus neutralization assay. Monitoring SARS-CoV-2 antibody responses induced by vaccination would provide important guidance for the optimization of COVID-19 vaccines.

## Background

Due to the ongoing coronavirus disease 2019 (COVID-19) pandemic, vaccination has been proved as the most effective means of alleviating or resolving the spread and outbreaks of the COVID-19 epidemic [1]. Full vaccination and booster vaccination can offer substantial protection against COVID-19 [2–4]. Large-scale vaccination against COVID-19 has been implemented in many countries as an exit strategy from unprecedented COVID-19-related restrictions. It has been established that a variety of vaccine strategies, including mRNA, viral vector, inactivated virus, and protein subunit, could effectively reduce symptomatic COVID-19 and further protect against severe and fatal diseases [5, 6]. Therefore, it is critical to closely monitor severe acute respiratory syndrome coronavirus 2 (SARS-CoV-2) specific antibody responses [7].

The evolution of multiple contagious SARS-CoV-2 variants was found with increased transmission or reduced efficacy of vaccines and therapeutics. Several variants have emerged from SARS-CoV-2 with alterations in their spike protein and other components in the viral genome, some of which have been identified as variants of concern (VOC) due to high transmission rates and disease severity [8]. The D614G (Ancestral) variant alters the conformation of the spike protein and accelerates the formation of an angiotensin-converting enzyme (ACE2)-binding fusion-competent state, thus enhancing virus infectivity [9]. Alpha (B.1.1.7), Beta (B.1.351), Gamma (P.1), and Delta (B.1.617.2), and Omicron (B.1.1.529) are designated as VOCs that have high frequency of mutations or deletions in the receptor binding domain (RBD) and N-terminus of the spike protein [10, 11]. Since these mutations could change interactions with the host receptor ACE2, thereby leading to reduced neutralization activities elicited by COVID-19 vaccines [12–14]. CoronaVac (Sinovac Biotech, Beijing, China), an inactivated, whole-virion COVID-19 vaccine, is the world’s most-used COVID-19 vaccine, which accounts for more than 2.5 billion vaccine doses delivered globally so far [15]. In addition, more than 200 million doses of other inactivated vaccines have been delivered, including China’s BBIBP-CorV from Sinopharm, India’s Covaxin, Iran’s OCVIran Barekat etc. [16]. Therefore, it is essential to have a deep understanding regarding longitudinal humoral responses and neutralizing antibody responses elicited by inactivated COVID-19 vaccines.

Starting from February 2021, a prospective, observational study was conducted in Nanjing Drum Tower Hospital, China to monitor dynamic SARS-CoV-2 specific IgM, IgG and surrogate neutralizing antibody (NAb) responses induced by CoronaVac, so as to provide a basis for optimization of vaccination strategy. Additionally, despite the pseudovirus neutralization assay is the predominant approach to quantify NAb levels, it is labor intensive and time-consuming in a relative low throughput manner, which is not suitable for clinical routine testing. Thereby, we also analyzed whether surrogate NAb levels determined by the authorized chemiluminescence assays could correlate with the NAb titers derived from pseudovirus neutralization assay.

## Results

### Demographic characteristics of the vaccine cohort

A total of 93 participants were enrolled in this vaccine cohort study, including 33 males (35.5%) and 60 females (64.5%). The median age of participants was 34 years old (IQR 28–40). Among them, the participants aged 21–30, 31–40, 41–50, and 51–60 were 34 (36.6%), 36 (38.7%), 15 (16.1%), and 8 (8.6%), respectively. The frequency of comorbidities is 2.1% (2/93) of participants, where 2 participants had hypertension. Besides, we have two additional convalescent cohorts. For convalescents cohort 1, 15 patients contracted SARS-CoV-2 in February 2020. In convalescents cohort 2, 5 individuals had breakthrough infections in February 2022, which was fully immunized with 2-dose CoronaVac during the period of May, 2021 and Sep 2021 (Table 1).

**Table 1:** Baseline characteristics of study population

Variables	CoronaVac vaccines (n = 93)	Convalescent cohort 1 (n = 15)	Convalescent cohort 2 (n = 5)
Age (years, median (P25, P75))	34 (28, 40)	50 (28, 59)	30 (27, 31)
Age group (years, n, %)			
21–30	34 (36.6%)	3 (20.0%)	2 (40.0%)
31–40	36 (38.7%)	3 (20.0%)	3 (60.0%)
41–50	15 (16.1%)	3 (20.0%)	0 (%)
51–60	8 (8.6%)	6 (40.0%)	0 (%)
Gender (n, %)			
Male	33 (35.5%)	7 (46.7%)	1 (20.0%)
Female	60 (64.5%)	8 (53.3%)	4 (80.0%)
Interval between 1st dose and 2nd dose (days)			
Median (IQR)	21 (17.3, 22.4)	NA	45 (42.8, 47.2)
Interval between 2nd dose and 3rd dose (months)			
Median (IQR)	8.6 (8.1, 8.7)	NA	NA
Seroconversion (%)			
IgG after 2 doses	97.8%	100%*	NA
Surrogate Nab after 2 doses	98.9%	100%*	NA
IgG after 3 doses	100%	NA	100%*
Surrogate Nab after 3 doses	100%	NA	100%*
Comorbidities (n, %)	2 (2.1%)	0 (%)	0 (%)

*Indicates the seroconversion of convalescents

### Seroconversion of SARS-CoV-2 antibodies

**Fig. 1:**
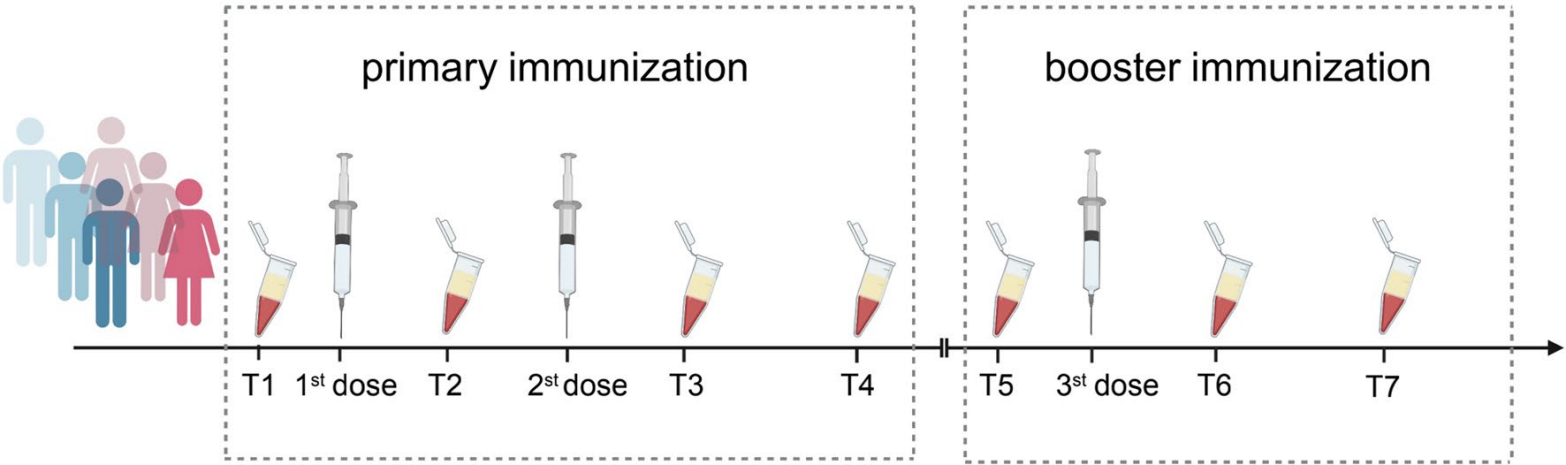
Schematic diagram of subject receiving 3-doses of inactivated COVID-19 vaccines and serum samples collection. COVID-19 inactivated vaccine inoculation is mainly divided into two phases, one is the primary immunization (left), the other is booster immunization (right). The serum samples of participants were collected at seven time points: before receiving first vaccine dose (Time point 1, T1), the 14th day after receiving the first dose of vaccination (Time point 2, T2), the 14th day after the second dose (Time point 3, T3), the 56th day after the second dose (Time point 4, T4), baseline before receiving the third dose (Time point 5, T5), the 14th day after the third dose (Time point 6, T6). and the 56th day after the third dose (Time point 7, T7)

Serum SARS-CoV-2-specific IgM, IgG, and surrogate neutralizing antibodies were measured at seven different time points, including baseline before the first dose, the 14th day after the first dose, the 14th day after the second dose, the 56th day after the second dose, baseline before the third dose, the 14th day after the third dose, and the 56th day after the third dose (Fig. 1). All participants were negative for three SARS-CoV-2 specific antibodies prior to vaccination. On the 14th day after the first dose of COVID-19 vaccine, 5.4% (5/93) of participants were positive for IgM antibodies in serum, 3.2% (3/93) were positive for IgG antibodies, and 24.7% (23/93) were positive for the surrogate NAb responses. Seropositivity rates of SARS-CoV-2 specific IgM, IgG, and surrogate NAb responses rose to 15.1% (14/93), 97.8% (91/93), and 98.9% (92/93), respectively, on the 14th day after the second dose. After 56 days of receiving the full immunization, SARS-CoV-2 specific IgM antibody titers rapidly declined to 1.1% (1/91), whereas seroconversion rates of SARS-CoV-2 specific IgG, surrogate NAb could be maintained at 91.3% (85/93). After 56 days of booster immunization, 100% (72/72) of participants were positive SARS-CoV-2 specific IgG antibodies and neutralizing antibody responses.

### Quantitative measurement of anti-SARS-CoV-2 humoral antibody responses

**Fig. 2:**
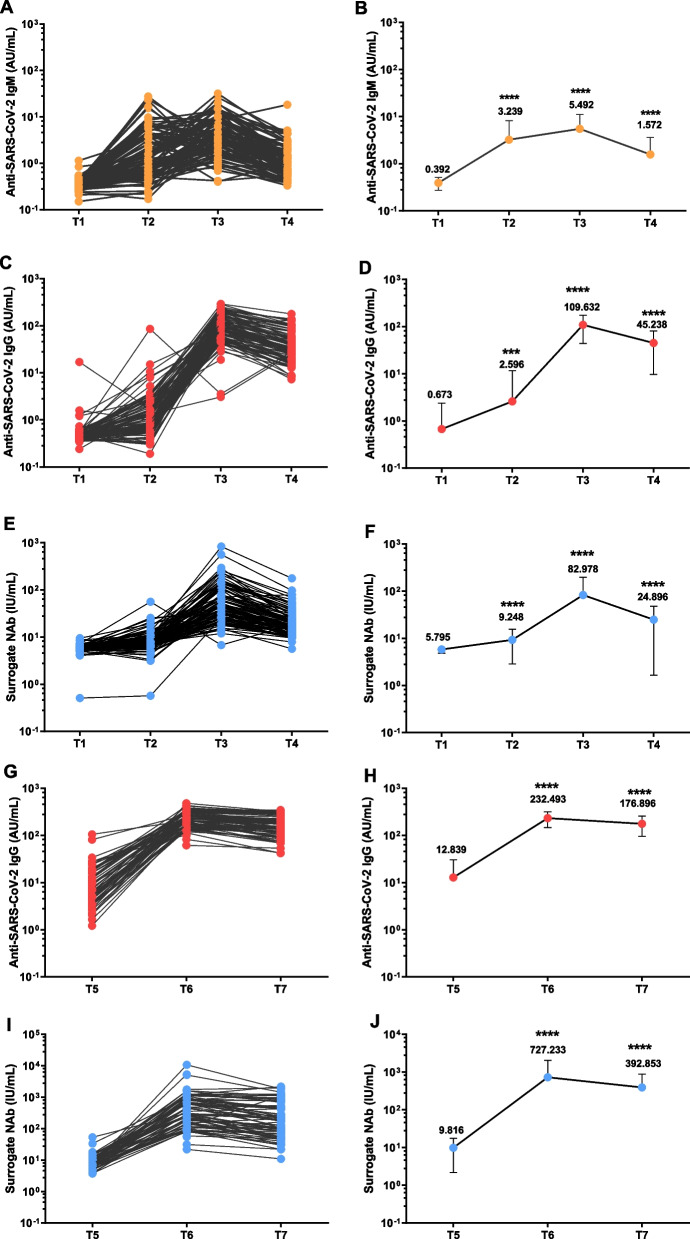
Quantification of SARS-CoV-2 specific antibodies induced by inactivated COVID-19 vaccines. The level of anti-SARS-CoV-2 IgM (**A**), IgG (**C**) and surrogate neutralizing antibodies (**E**) in 93 enrolled healthy participants from T1 to T4. The level of anti-SARS-CoV-2 IgG (**G**) and surrogate neutralizing antibodies (**I**) in 93 enrolled healthy participants from T5 to T7. The total levels of anti-SARS-CoV-2 IgM (**B**), IgG (**D**) and surrogate neutralizing antibodies (**F**) after primary immunization. The total levels of anti-SARS-CoV-2 IgG (**H**) and surrogate neutralizing antibodies (**J**) after booster immunization. Statistical significance was determined by Kruskal–Wallis test. *P < 0.05; **P < 0.01; ***P < 0.001; ****P < 0.0001

SARS-CoV-2 specific antibodies induced by CoronaVac were quantified at seven different time points (Fig. 2A, C, E, G, J). Prior to first dose (T1), baseline levels of SARS-CoV-2 specific IgM, IgG, and surrogate NAb were 0.38 (IQR 0.34–0.43) AU/mL, 0.47 (0.42–0.52) AU/mL, 5.76 (5.37–6.13) IU/mL, respectively (Fig. 2B, D, F). The first dose (T2) resulted in a rapidly increase of SARS-CoV-2 specific IgM, IgG, and surrogate NAb levels, 1.62 (0.63–3.84) AU/mL, 0.90 (0.59–1.82) AU/mL and 7.74 (6.41–9.94) IU/mL, respectively (Fig. 2B, D, F). The second vaccination further led to significant elevation of SARS-CoV-2 specific IgM (3.45, 1.82–7.03 AU/mL), IgG (91.21, 55.66–152.06 AU/mL), and surrogate neutralizing antibodies (47.60, 25.96–100.81 IU/mL) at T3 timepoint, compared to those at T2 timepoint (*P* < 0.0001) (Fig. 2B, D, F). Furthermore, SARS-CoV-2 specific IgM, IgG, and surrogate NAb titers at T4 timepoint were decreased compared to T3, 1.06 (0.65–1.84) AU/mL, 32.42 (20.41–55.18) AU/mL, and 18.10 (12.96–24.8) IU/mL (*P* < 0.001) (Fig. 2B, D, F). After 9 months, a booster immunization was conducted. SARS-CoV-2 specific humoral responses were continuously monitored before booster immunization, 14 days and 56 days after booster dose. The antibody titers of SARS-CoV-2 specific IgG at T5, T6, T7 were 6.46 (3.85–17.29) AU/mL, 218.29 (167.53–292.16) AU/mL, 172.87 (118.93–219.48) AU/mL, respectively; while the surrogate NAb at T5, T6, and T7 antibody titers were 7.66 (6.04–10.91) IU/mL, 445.54 (171.54, 810.90) IU/mL, 180.96 (69.48, 481.98) IU/mL, respectively. Both of SARS-CoV-2 specific IgG responses and surrogate NAb at T6 and T7 were significantly elevated more than tenfold compared to T5 (*P* < 0.05) (Fig. 2H, J).

### Effect of gender and age on humoral immune response to COVID-19 vaccines

**Fig. 3:**
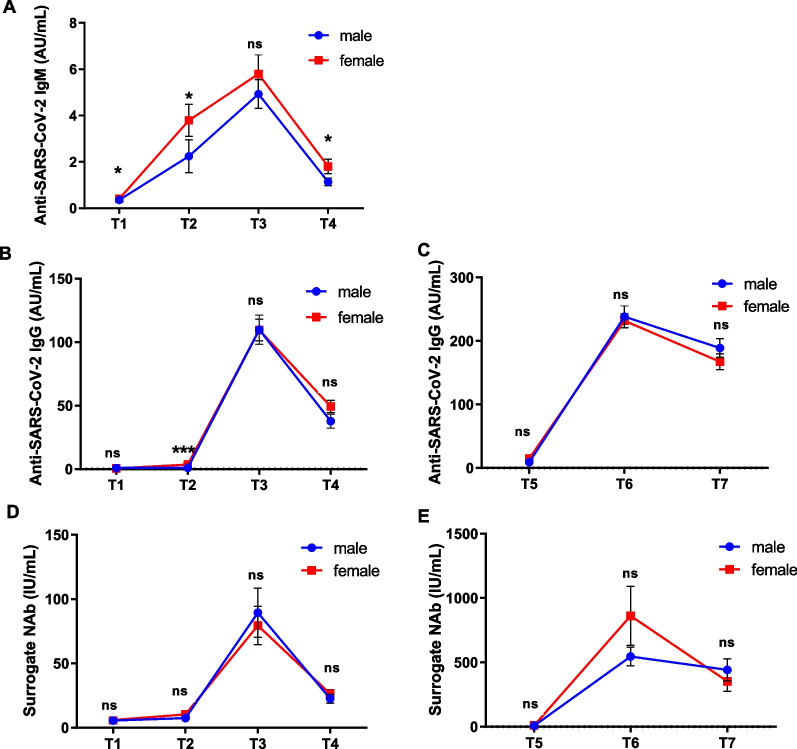
Effect of gender on humoral immune response induced by CoronaVac. **A**–**E** The levels of IgM, IgG, and surrogate neutralizing antibodies responses between males and females at the primary immunization phase (from T1 to T4) and the booster immunization phase (from T5 to T7). Statistical significance was determined by Mann–Whitney U-test. ns indicates no statically significance. *P < 0.05; ***P < 0.001

Previous studies suggested that female might mount stronger humoral immune responses to various types of vaccination [17]. To analyze the effect of gender on COVID-19 vaccine-induced humoral immune responses, we then compared antibody levels among females versus males (Fig. 3). Statistical results indicated that female participants mounted higher anti-SARS-CoV-2 IgM at T1, T2, and T4 stages than that of male participants (*P* < 0.05) (Fig. 3A). Males had significantly higher anti-SARS-CoV-2 IgG titers at T2 than females (*P* < 0.001) (Fig. 3B). Moreover, there was no significant differences between genders in terms of surrogate NAb induced by the COVID-19 vaccine (*P* > 0.05) (Fig. 3D, E).

**Fig. 4:**
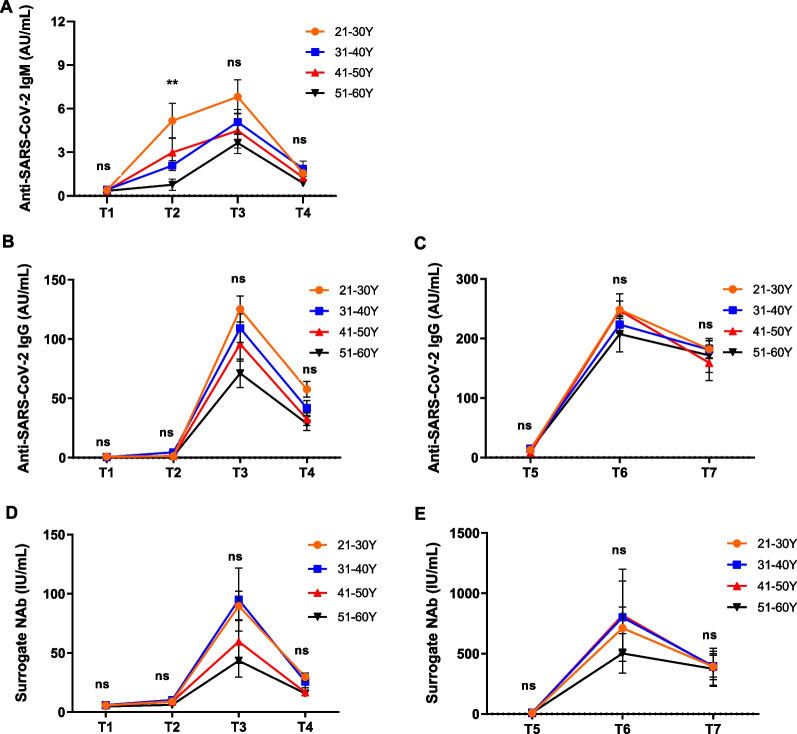
Effect of age on humoral immune response induced by CoronaVac. The levels of anti-SARS-CoV-2 IgM (**A**), IgG (**B**, **C**) and surrogate neutralizing antibodies (**E**, **F**) among participants of different age groups (21–30 years, 31–40 years, 41–50 years, 51–60 years) were compared. ns indicates no statically significance. Statistical significance was determined by Kruskal–Wallis test. ns indicates no statically significance. **P < 0.01

Since aging might influence COVID-19 severity and response to vaccine [18–20], we further explored the impact of age on the humoral immune responses elicited by CoronaVac (Fig. 4). After the first dose, SARS-CoV-2 specific IgM antibody levels in participants aged 21–30 (1.95, 0.86–0.96 AU/mL) were remarkably higher than those in participants 51–60 (22.76, 22.15–75.91 AU/mL) at T2 timepoint (*P* < 0.01) (Fig. 4A). Our data indicate no significant differences in SARS-CoV-2 specific IgM, IgG, and surrogate NAb levels among different age groups at other stages of primary immunization or booster vaccination (*P* > 0.05) (Fig. 4A–E). Altogether, our results indicated that participants aged between 21 and 30 years old could rapidly mount humoral immune response to CoronaVac compared with those aged 51–60 years old.

### Correlation analysis of neutralizing antibody responses determined by the pseudovirus neutralization assay and the chemiluminescence assays

**Fig. 5:**
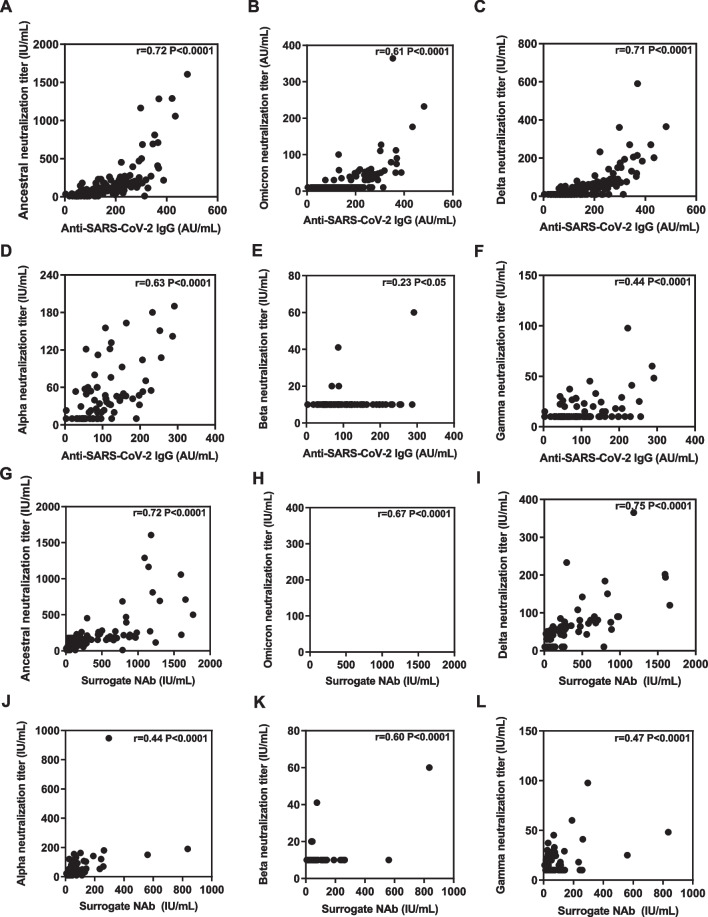
Correlations between neutralizing antibody responses of SARS-CoV-2 variants and antibodies induced by vaccination. **A**–**L** Scatter plot of pairwise correlation between anti-SARS-CoV-2 IgG, surrogate neutralizing antibodies and neutralizing antibodies against SARS-CoV-2 variants Alpha, Gamma, Beta, Omicron, Delta, Ancestral. The r value was calculated by Pearson correlation analysis

Using standard pseudovirus neutralization assay, we previously reported neutralizing antibodies against SARS-CoV-2 wildtype strain as well as and emerging variants using our cohort [12, 18, 21]. To determine whether the surrogate NAb responses tested by the chemiluminescent assay could represent the level of neutralization titers determined by pseudovirus neutralization assay, correlation analyses for results derived from two assays were performed. We found strong positive correlation between the magnitude of anti-SARS-CoV-2 IgG responses and neutralizing antibodies against Ancestral strain (r = 0.72, *P* < 0.0001), Omicron strain (r = 0.61, *P* < 0.0001), Delta strain (r = 0.71, *P* < 0.0001), Alpha strain (r = 0.63, *P* < 0.0001), respectively (Fig. 5A–D). Similarly, significant positive correlations were also observed between SARS-CoV-2 surrogate NAb and neutralizing antibodies against the Ancestral (r = 0.72, *P* < 0.0001), Omicron (r = 0.67, *P* < 0.0001), Delta (r = 0.75, *P* < 0.0001), Beta (r = 0.60, *P* < 0.0001) variants, respectively (Fig. 5G, H, I, K).

### The comparison of antibody responses between vaccinated individuals and COVID-19 convalescent patients

**Fig. 6:**
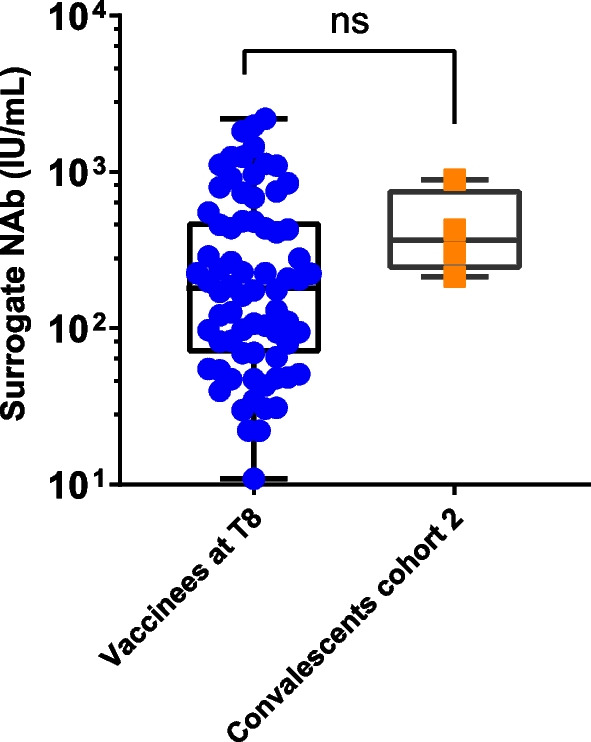
Antibody activity of convalescent serum from COVID-19 recovery patients. **A**, **B** Comparison of anti-SARS-CoV-2 IgG and surrogate neutralizing antibody levels at T4 phase serum from 93 healthy volunteers received vaccination and serum from 15 individuals who contracted SARS-CoV-2 in February 2020 (convalescents cohort 1). **C**, **D** Comparison of anti-SARS-CoV-2 IgG and surrogate neutralizing antibody levels at T7 phase serum from 93 healthy volunteers received vaccination and serum from 5 individuals who had breakthrough infections in February 2022 (convalescents cohort 2) Statistical significance was determined by Mann–Whitney U-test. ns indicates no statically significance. **P < 0.01

To compare the level of antibody responses between vaccinated individuals and COVID-19 convalescent patients, the SARS-CoV-2 IgG and surrogate NAb levels were also measured in convalescent serum from COVID-19 patients, including 15 individuals who were contracted SARS-CoV-2 in year 2020 and 5 individuals who had breakthrough infection after full immunization in year 2022. We found that anti-SARS-CoV-2 IgG and surrogate NAb responses from serum collected from 8 to 16 weeks after COVID-19 infection in the year 2020 were comparable with those vaccinated serum collected at T4. (Fig. 6A, B). Nevertheless, the breakthrough infection results in remarkable higher level of anti-SARS-CoV-2 IgG antibody responses when compared to the serum from 3-dose CoronaVac recipients collected at T7 (Fig. 6C), suggesting that breakthrough infection could induce significantly higher humoral responses than booster dose (*P* < 0.01).

## Discussion

Vaccination with CoronaVac showed high efficacy against PCR-confirmed symptomatic COVID-19, hospital admissions, and fatality during Gamma [22], Delta [23], and Omicron [24] variants associated of COVID-19 epidemic. The vaccination also showed a good safety and tolerability profile expanded to the adults aged 60 years and older as well as children and adolescents aged 3–17 years [25–27]. Therefore, it is critical to have a detailed understanding of the kinetics and magnitude of humoral responses elicited by three-dose CoronaVac.

Using the highly sensitive chemiluminescent assay for SARS-CoV-2 specific humoral responses, our data showed that the CoronaVac was highly immunogenic and could induce protective antibodies against SARS-CoV-2 [28]. The seroconversion rate of SARS-CoV-2 specific IgG antibody reached as high as 91.3% and 100% when the participants completed the second dose and third dose of vaccination, respectively. Since not all SARS-CoV-2 specific antibodies have neutralization potential [29], IgG, IgA, and IgM antibodies which competitively bind to ACE2 with RBD were defined as surrogate neutralizing antibodies in our assays, accounting for the major proportion of SARS-CoV-2 protective antibodies which can prevent the viral entry into infected cells [30]. The seroconversion rate of surrogate NAb also reached 91.3% and 100% after 2 doses and 3 doses of vaccination, respectively. Nevertheless, we also found that the breakthrough infection was more effective to induce anti-SARS-CoV-2 IgG antibody responses than the booster dose.

SARS-CoV-2 specific IgM, IgG and surrogate NAb across different age groups were also quantitatively analyzed in our cohort. Notably, volunteers aged between 21 and 30 years mounted significantly higher level of SARS-CoV-2 specific IgM than volunteers aged between 51–60 years old after receiving 2 doses of CoronaVac, consistent with previous observation that elder individuals might have an impaired immune response to SARS-CoV-2 infection and vaccine [31]. Notably, there was no significant difference observed in IgG and surrogate NAb titers between age groups after booster dose, suggesting that the third dose of CoronaVac is highly immunogenic and is beneficial to improve the vaccine protectivity especially for individuals aged over 50 years old [25, 32]. Consistent with our findings, data from a recent population-based observational study in Hongkong showed that 2-dose and 3-dose of CoronaVac offered 69.9% and 97.9% of protection against severe and fatal outcome, respectively [24], suggesting that the full vaccination of CoronaVac provide the satisfactory protection, possibly via the protective antibody responses induced by the vaccine.

SARS-CoV-2 neutralizing antibodies are one of the most important laboratory parameters to evaluate the vaccine efficacy, which could protect against SARS-CoV-2 infection by preventing viral entry into target cells [33]. The gold standard for detecting the blocking antibodies is to directly detect whether the serum antibody can inhibit live viruses or pseudoviruses from entering into cells that express ACE2 receptor, but this method is relatively cumbersome, requiring 2–4 days to complete the detection, and is not suitable for clinical large-scale application [34]. Herein, the surrogate NAb in our study used the competitive binding approach which evaluated the antibodies could block RBD protein binding to ACE2, which could be performed in routine clinical laboratories in a high-throughput manner. Indeed, the magnitude of surrogate NAb response was highly correlated with neutralizing antibodies against all circulating SARS-CoV-2 VOCs, including Ancestral, Alpha, Beta, Gamma, Delta and Omicron strains. We also recognized that some special neutralizing antibodies targeting other region on the spike protein such as N-terminal domain (NTD) [35] and fusion peptide [36] were not included in this assay. Nevertheless, our data highlighted that the surrogate NAb test is helpful to predict the protective immune responses elicited by CoronaVac vaccine.

Our study has several limitations. First, our cohort only included the vaccine recipients who received homologous boosting of CoronaVac. Unfortunately, those who received previous 2-dose CoronaVac and boosted with heterologous vaccines such as mRNA vaccines or protein subunit-based vaccines were not included. Previous studies suggested that despite CoronaVac induced relative lower neutralizing activity against VOCs than natural infection as well as mRNA-based vaccines such as BNT162b2 [37, 38]. It will be interesting to directly compare the immune responses across different vaccine platforms and schedules. Second, either vaccine recipients or COVID-19 convalescents might also experience rapidly waned humoral responses especially for neutralization activities [29, 38, 39]. Here we only followed up the humoral responses 56 days after the second and third dose of CoronaVac. The longer follow-up is still needed to assess the duration of antibody responses elicited by CoronaVac.

## Conclusion

CoronaVac is highly immunogenic and could induce SARS-CoV-2 specific IgG and NAb responses after two doses of vaccination, whereas the third dose of CoronaVac is necessary to further boost the suboptimal SARS-CoV-2 specific humoral responses, particularly in elder adults. Our data has immediate implication for multiple countries that previously used a CoronaVac regimen, which may guide the optimization of vaccine strategy to combat COVID-19 pandemic.

## Methods

### Study cohort

A prospective cohort study was carried out among 93 healthy individuals from Drum Tower Hospital who had completed 3 doses of SARS-CoV-2 vaccination between January 28, 2021 and Nov 31, 2021. The first two doses were administered from Jan 27th,2021 to March 15th, 2021, and median of two-dose interval was 21 days. The booster immunization was performed from Nov 8th, 2021 to Nov 12th, 2021. All participants signed informed consents. Those with previous history of COVID-19 infection and contraindications for SARS-CoV-2 vaccination were excluded. We also collected convalescent serum from 15 patients contracted SARS-CoV-2 in February 2020 (convalescents cohort 1). In addition, we enrolled 5 individuals who had breakthrough infections in February 2022 and collected their serum in May 2022 (convalescents cohort 2) (Table 1). This study has been registered with ClinicalTrials.gov (NCT04729374) on Jan 28th, 2021 and has been approved by the Ethics Committee of Nanjing Drum Tower Hospital (2021-034-01). All the methods were carried out in accordance with relevant guidelines.

### Serum collection

Blood samples were collected from each individual at seven time points: before receiving first vaccine dose (timepoint 1, T1), the 14th day after receiving the first dose of vaccination (Timepoint 2, T2), the 14th day after the second dose (Timepoint 3, T3), the 56th day after the second dose (Timepoint 4, T4), baseline before receiving the third dose (Timepoint 5, T5), the 14th day after the third dose (Timepoint 6, T6), and the 56th day after the third dose (Time point 7, T7). In addition, 20 convalescent sera were collected, in which 15 serum samples were collected between 8 and 16 weeks from COVID-19 convalescent patients who contracted SARS-CoV-2 in February 2020, and 5 serum samples were collected at the week 8 after breakthrough infections in February, 2022. Serum was separated by centrifugation for 10 min at 2000×*g* at room temperature in Allegra X-15R Centrifuge (Beckman Coulter, USA), and stored at − 80℃.

### The measurement of SARS-CoV-2 specific IgM, IgG and surrogate neutralizing antibody responses

SARS-CoV-2 specific IgM and IgG antibodies were measured by a two-step indirect immunoassay using the iFlash3000-C Chemiluminescence Immunoassay Analyzer (Shenzhen Yhlo Biotech Co., Ltd, China). SARS-CoV-2 surrogate neutralizing antibodies were determined by a one-step immune-competition assay by measuring total antibody levels (IgM, IgG, and IgA) in serum that could compete with SARS-CoV-2 spike protein receptor binding region (RBD) for binding to SARS-CoV-2 receptor, angiotensin-converting enzyme II (ACE2). The negative reference range for SARS-CoV-2 specific IgM and IgG antibodies was 0–10 AU/mL. The negative reference range for SARS-CoV-2 surrogate neutralizing antibodies was 0–10 IU/mL.

### Pseudovirus neutralization assay

Pseudovirus neutralization assay was performed as previously described [7] to detect neutralization titers. Lentivirus-based SARS-CoV-2 pseudovirus (Vazyme Biotech Co, Ltd., China) was generated by co-transfection of an HIV-1 NL4-3 luciferase reporter vector containing defective Nef, Env, and Vpr (pNL4-3.luc.RE) and a pcDNA 3.1(Invitrogen, USA) expression plasmid encoding the corresponding spike protein (D614G, Alpha, Beta, Delta, and Omicron). The luciferase relative light unit method (RLU) was performed to detect the 50% tissue culture infectious dose (TCID50) of SARS-CoV-2 pseudovirus. Neutralizing antibody data determined by pseudovirus neutralization assay was previously published [12, 21].

### Statistical analysis

The data analysis was conducted by SPSS 22.0 software. The numeric variables were described as frequency or percentage. Normally distributed numeric data was described by mean ± standard error and non-normally distributed numeric data was expressed by median and quartiles [M (P25, P75)]. The independent group t test (normal distribution), Mann–Whitney U (non-normal distribution) were used to compare continuous variables between groups.one-way ANOVA (normal distribution) and Kruskal–Wallis (normal distribution) were used to compare continuous variables among groups. All graphical representations were conducted by GraphPad Prism 9.0 software. *P* value < 0.05 was considered statistically significant.

## Data Availability

The datasets used and analysed during the current study available from the corresponding author on reasonable request.

## Abbreviations

COVID-19: Coronavirus disease 2019
SARS-CoV-2: Severe acute respiratory syndrome coronavirus 2
ACE2: Angiotensin-converting enzyme II
RBD: Receptor binding region
VOC: Variant of concern
NTD: N-terminal domain
NAb: Neutralizing antibody
